# Anticoagulation management for cardiopulmonary bypass using TEG® 6 s in a patient receiving both heparin and dabigatran

**DOI:** 10.1186/s40981-024-00739-8

**Published:** 2024-09-04

**Authors:** Yu Kawada, Nobuyuki Katori, Keiko Kaji, Shoko Fujioka, Tomoki Yamaguchi

**Affiliations:** Department of Anesthesiology, The Jikei University School of Medicine, Tokyo, 105-8461 Japan

**Keywords:** Thromboelastography, Heparinase, Activated clotting time, Dabigatran, Heparin, Cardiopulmonary bypass

## Abstract

**Background:**

It is difficult to evaluate adequate dose of heparin for cardiopulmonary bypass (CPB) by activated clotting time (ACT) in a patient receiving both heparin and dabigatran because dabigatran can also prolong ACT. We evaluated the effect of dabigatran by thromboelastography (TEG) to determine adequate heparin dose for CPB.

**Case presentation:**

An 81-year-old woman receiving both heparin and dabigatran was scheduled for an emergency surgical repair of iatrogenic atrial septal perforation. Although ACT was prolonged to 419 s, we performed TEG to distinguish anticoagulation by dabigatran from heparin comparing R in CK and CHK. As the results of TEG indicated residual effect of dabigatran, we reversed dabigatran by idarucizumab and then dosed 200 U/kg of heparin to achieve adequate anticoagulation for CPB by heparin.

**Conclusions:**

TEG could help physicians to determine need for idarucizumab and also an adequate dose of heparin to establish appropriate anticoagulation for CPB.

## Background

The standard agent for systemic anticoagulation during cardiopulmonary bypass (CPB) is unfractionated heparin (UFH), which is titrated with monitoring of activated clotting time (ACT). Direct thrombin inhibitors (DTIs) such as argatroban and bivalirudin are alternatives to UFH, which are sometimes used in patients having contraindications to heparin use. However, there have been concerns about inadequate anticoagulation with DTI resulting in clotting in the extracorporeal circuit [1–3], despite ACT being maintained above 480 s which is recognized as the minimum acceptable value for adequate anticoagulation during CPB [4]. Thus, it would be better to distinguish the effect of UFH from that of DTI to determine an adequate dose of UFH for CPB management in a patient receiving both UFH and DTI. However, ACT is not suitable to evaluate the effect of UFH in such a case because DTIs also prolong ACT.

We, hereby, report an emergency cardiac case in which we evaluated the anticoagulation effect of dabigatran, an oral DTI, by thromboelastography to determine an adequate dose of UFH for CPB in a patient who had received both UFH and dabigatran.

## Case presentation

An 81-year-old woman with a height of 140 cm and weight of 42 kg was scheduled for percutaneous transluminal septal myocardial ablation for persistent atrial flutter. The patient took 110 mg of dabigatran on the morning of the ablation and received a continuous infusion of UFH during the procedure. ACT was 323 s before heparinization and maintained at more than 400 s during the procedure. The patient was sedated with a continuous infusion of propofol and received oxygen inhalation via a face mask during the procedure. The procedure was continued as scheduled, although her SpO2 showed a trend of decreasing. SpO2 indicated 80–85% despite 15 L/min oxygen inhalation via a face mask at the end of the procedure. Her trachea was intubated, and the examination by a contrast-enhanced CT and echocardiography proved a right-left shunt in the atrial septum owing to septal perforation. After placing a balloon occlusion catheter at the atrial septal perforation site, the patient was transferred to the OR for an emergency surgical repair of iatrogenic atrial septal perforation.

**Fig. 1:**
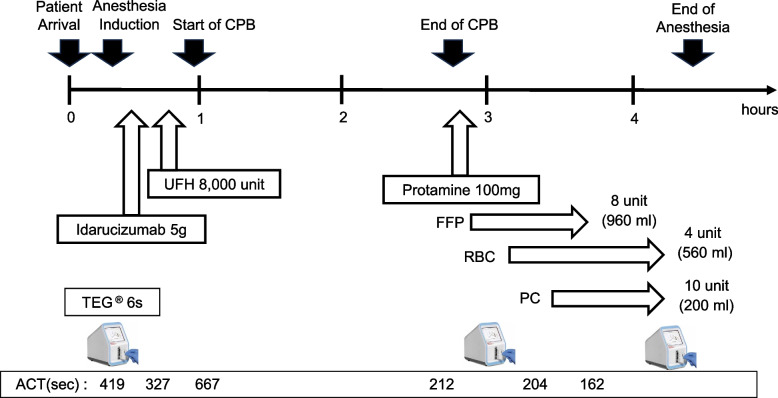
Anesthesia course. Abbreviations: CPB, cardiopulmonary bypass; UFH, unfractionated heparin; FFP, fresh-frozen plasma; RBC, red blood cells; PC, platelet concentrate; ACT, activated clotting time

ACT after anesthesia induction was 419 s, which seemed fair for the initiation of CPB (Fig. 1). We also performed the global hemostasis test on thromboelastography (TEG® 6 s, Haemonetics®, Braintree, MA, USA) to evaluate the anticoagulation by dabigatran and UFH. Global hemostasis test can perform four kinds of assays simultaneously: CK assay activated by kaolin, CKH assay activated by kaolin in combination with heparinase, CRT assay activated by kaolin and tissue factor, and CFF assay corresponding to plasma fibrinogen level. Among assays included in the global hemostasis test, we evaluated the reaction time (R), corresponding to thrombin generation time like APTT, in CK and CKH assays. As CK assay activates coagulation by kaolin, CK-R is affected by both UFH and dabigatran. Although CKH also activates coagulation by kaolin, it includes heparinase which abrogates the effect of UFH. Thus, the prolongation of CKH-R indicates the presence of an anticoagulant other than UFH, meaning dabigatran in our case. CKH-R was prolonged by 10 min over the upper limit of the normal range (Table 1), indicating the effect of dabigatran was considerable.

**Table 1:**
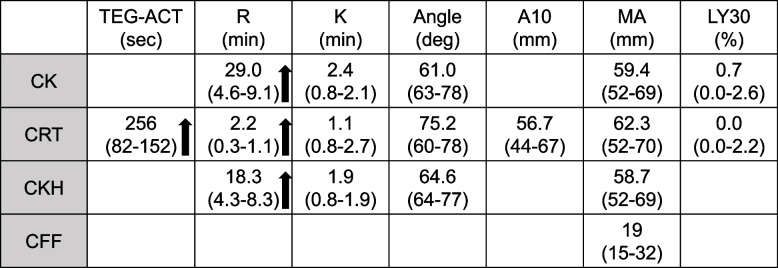
Results of TEG after anesthesia induction

Five grams of idarucizumab was dosed to reverse the effect of dabigatran, and we found ACT was 327 s after idarucizumab infusion. After confirmation of the effect of residual heparin, we administered 8000 units (200 U/kg) of UFH before the CPB initiation (Fig. 1). ACT reached 677 s after the bolus of UFH and was maintained at more than 480 s during CPB. The patient underwent direct shunt closure, tricuspid valve plication, and left atrial appendage closure. After the separation from CPB, we administered 100 mg of protamine and found ACT was 212 s (Fig. 1). We also performed TEG and found the ratio of CK-R to CKH-R was almost 1.0 indicating the adequate reversal of UFH (Table 2). However, we considered thrombin generation was aggravated because both TEG-ACT and CKH-R were prolonged (Table 2). We also considered both fibrinogen platelet replacement would be necessary because MA in CK, CRT, and CFF assays did not reach the lower limit of normal ranges: decrease in MA was more profound in CK and CRT compared to CFF. Adequate hemostasis was achieved after the transfusion of 960 mL of fresh-frozen plasma, 200 mL of platelet concentrates, and packed red blood cells. The results of TEG after transfusions indicated recovery of coagulation (Table 3.), and the patient was transferred to the ICU.

**Table 2:**
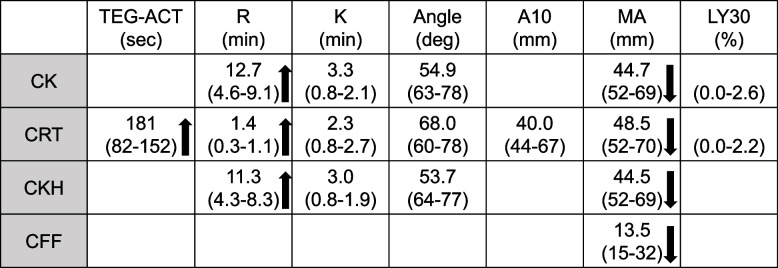
Results of TEG after protamine

**Table 3.** Results of TEG at the end of surgery

	TEG-ACT (s)	R (min)	K (min)	Angle (deg.)	A10 (mm)	MA (mm)	LY30 (%)
CK		8.2 (4.6–9.1)	1.7 (0.8–2.1)	68.0 (63–78)		58.6 (52–69)	1.1 (0.0–2.6)
CRT	97.3 (82–152)	0.5 (0.3–1.1)	1.6 (0.8–2.7)	74.1 (60–78)	52.2 (44–67)	58.9 (52–70)	0.6 (0.0–2.2)
CKH		11.2 (4.3–8.3)	2.3 (0.8–1.9)	63.6 (64–77)		57.9 (52–69)	
CFF						19 (15–32)	

Normal ranges for each parameter are shown in parentheses

*CK* kaolin assay, *CRT* kaolin and tissue factor assay, *CKH* kaolin with heparinase assay, *CFF* functional fibrinogen assay, *A10* amplitude at 10 min after confirmation of reaction time, *MA* maximum amplitude, *R* reaction time, *K* kinetics, *MA* maximal amplitude, *LY30* clot lysis at 30 min after maximum clot strength

## Discussion

ACT is the standard monitor to evaluate anticoagulation by UFH in cardiac surgery requiring CPB. Although it is recommended to maintain ACT at more than 480 s during CPB [4], the threshold of 480 s has been developed only for UFH; the recommended ACT threshold for DTI or the combination of UFH and DTI has not been established. Indeed, intravenous DTI such as argatroban or bivalirudin is recommended as an alternative to UFH in a patient with heparin contraindications [4, 5], and the quality of anticoagulation with DTI may differ from that with UFH because several case reports indicated thrombus formation in the CPB circuit, although ACT showed a prolonged value of more than 480 s during CPB [1–3]. The difference between DTI and UFH in the risk of thrombus formation during CPB may be attributed to a characteristic of DTI inhibiting the initiation phase in coagulation but not the propagation and amplification phases [6, 7]. Furthermore, anticoagulation with dabigatran, an oral DTI, during CPB is still under exploration for future clinical use, and its safety in the clinical setting is not confirmed [8, 9]. Thus, we considered that anticoagulation in our case should be established by an adequate dose of UFH even though ACT was prolonged partially by dabigatran. However, we could not determine the dose of UFH according to ACT because ACT was prolonged due to both dabigatran and UFH. To evaluate the anticoagulation by dabigatran and UFH separately, we compared R between CK and CKH assays in TEG. As described in the case presentation, the prolongation of CKH-R indicates the effect of dabigatran because heparinase contained in the CKH reagent abrogates the effect of UFH. CKH-R showed a prolonged value of 18.3 min (normal range: 4.3–8.3), which corresponds to a plasma dabigatran concentration in the therapeutic range according to the results of an in vitro study investigating the effects of dabigatran on TEG [10].

We considered significant amount of dabigatran was contributing to prolonged ACT and administered idarucizumab to abrogate the effect of dabigatran so that we could evaluate the effect of UFH on ACT. Idarucizumab is the specific antidote for dabigatran and is approved for preemptive administration before an urgent surgery in which severe bleeding due to dabigatran is anticipated. Idarucizumab specifically binds to dabigatran with high affinity and instantly abrogates the anticoagulant activity of dabigatran without affecting the effect of UFH. It does not bind to thrombin substrates including factors V, VIII, and XIII or fibrinogen, nor exhibit thrombin-like activity, as demonstrated by a lack of activity in coagulation or platelet aggregation tests [11]. Thus, idarucizumab does not directly activate coagulation system, instead, it works by neutralizing dabigatran, which indirectly leads to the normalization of coagulation parameters such as R in TEG.

After administration of idarucizumab, ACT decreased from 419 to 327 s, indicating insufficient anticoagulation by UFH for the initiation of CPB. Although it was uncertain about the exact dose of UFH to increase ACT from 327 s to more than 480 s, we administered 200 U/kg of UFH based on our clinical experience and increased ACT more than 600 s. One of the advantages of performing TEG in a patient receiving both UFH and dabigatran is to facilitate the determination of the indication for idarucizumab. As described previously, anticoagulation by DTI during CPB should be avoided so far as possible due to the risk of thrombus formation. Indeed, it might seem routine administration of idarucizumab before heparinization for CPB could be reasonable in such a patient; however, the cost of idarucizumab, more than US $5000 for 5 g, does not always allow the routine dosing. Ecarin clotting time (ECT) is one of the specific assays for monitoring the effect of DTI such as dabigatran [12]; however, it is not a common laboratory test in clinical practice. CK-R in TEG which is a coagulation time activated by kaolin has been shown to correlate with plasma dabigatran concentration and could be a surrogate for ECT [13]. As the effect of UFH is abrogated by heparinase in CKH assay, prolongation of CKH-R could be a parameter to evaluate the effect of residual dabigatran. To avoid the unnecessary administration of idarucizumab, performing TEG in a patient receiving both UFH and dabigatran, especially evaluating CK-R and CHK-R, would be helpful to physicians.

There are two limitations in this case report. The first is the ability of heparin neutralization by heparinase used in CKH assay. As UFH activity could be accurately quantified by the ratio of CK-R to CKH-R in the range of 0.05 to 0.8 U/mL [14], the effect of dabigatran might be overestimated in case the heparin concentration is more than 0.8 U/mL. However, the concentration between 0.3 and 0.7 U/mL is recommended as a therapeutic range for the treatment of venous thromboembolism with UFH [15]. And it has been shown that the range for therapeutic vascular procedures including aortic endovascular surgery is between 0.5 and 1.0 U/mL [16]. Thus, we consider TEG could be applicable even if the patient received UFH in antithrombotic therapy or most vascular procedures requiring low-dose UFH. The second is the threshold of CKH-R prolongation to determine the administration of idarucizumab is not defined clearly. It has been shown that dabigatran could prolong ACT and CK-R in a dose-dependent manner [9]. And these parameters went up over the upper limit of the normal range at 50 ng/mL of dabigatran, which is slightly lower than the trough level of dabigatran (70–90 ng/mL) [17]. Thus, we speculate idarucizumab should be administered to minimize the effect of dabigatran on ACT when CKH-R exceeds the normal range.

In conclusion, global hemostasis test in TEG, especially comparing CK-R and CKH-R, could be useful to evaluate the residual effect of dabigatran in a patient receiving both UFH and dabigatran. It could help physicians to determine the need for idarucizumab and also an adequate dose of UFH to establish appropriate anticoagulation for CPB.

## Data Availability

Not applicable.

## Abbreviations

CPB: Cardiopulmonary bypass
UFH: Unfractionated heparin
ACT: Activated clotting time
DTI: Direct thrombin inhibitor
